# Antivenom Treatment Is Associated with Fewer Patients using Opioids after Copperhead Envenomation

**DOI:** 10.5811/westjem.2019.3.42693

**Published:** 2019-04-26

**Authors:** Caroline E. Freiermuth, Eric J. Lavonas, Victoria E Anderson, Kurt C. Kleinschmidt, Kapil Sharma, Malin Rapp-Olsson, Charles Gerardo, Copperhead Recovery Workgroup, Sean Bush, Michael Mullins, Spencer Greene, Eric Toschlog, Eugenia Quackenbush, S. Rutherford Rose, Richard Schwartz, Nathan Charlton, Brandon Lewis, Nicklaus Brandehoff

**Affiliations:** * Duke University, Division of Emergency Medicine, Durham, North Carolina; † Denver Health and Hospital Authority, Rocky Mountain Poison and Drug Center, Denver, Colorado; ‡ University of Texas Southwestern Medical Center, Department of Emergency Medicine, Dallas, Texas; 1 Brody School of Medicine, Department of Emergency Medicine, Greenville, North Carolina; 2 Washington University School of Medicine, Division of Emergency Medicine, St. Louis, Missouri; 3 Baylor College of Medicine, Department of Emergency Medicine, Houston, Texas; 4 Brody School of Medicine, Department of Surgery, Greenville, North Carolina; 5 University of North Carolina, Department of Emergency Medicine, Chapel Hill, North Carolina; 6 Virginia Commonwealth University, Department of Emergency Medicine, Richmond, Virginia; 7 Medical College of Georgia, Department of Emergency Medicine and Hospital Services, Augusta, Georgia; 8 University of Virginia, Department of Emergency Medicine, Charlottesville, Virginia; 9 Texas A&M Health Science Center, Department of Emergency Medicine, College Station, Texas; 10 Rocky Mountain Poison and Drug Center, Denver, Colorado

## Abstract

**Introduction:**

Copperhead envenomation causes local tissue destruction, leading people to seek treatment for the pain and swelling. First-line treatment for the pain is opioid medications. There is rising concern that an initial opioid prescription from the emergency department (ED) can lead to long-term addiction. This analysis sought to determine whether use of Fab antivenom (FabAV) for copperhead envenomation affected opioid use.

**Methods:**

We performed a secondary analysis using data from a randomized clinical trial designed to determine the effect of FabAV on limb injury recovery following mild to moderate copperhead envenomation. Opioid use was a defined secondary outcome in the parent trial. Patients were contacted after discharge, and data were obtained regarding medications used for pain and the patients’ functional status. This analysis describes the proportion of patients in each treatment group reporting opioid use at each time point. It also assesses the interaction between functional status and use of opioids.

**Results:**

We enrolled 74 patients in the parent trial (45 received FabAV, 29 placebo), of whom 72 were included in this secondary analysis. Thirty-five reported use of any opioids after hospital discharge. A smaller proportion of patients treated with FabAV reported opioid use: 40.9% vs 60.7% of those in the placebo group. The proportion of patients using opioids remained smaller in the FabAV group at each follow-up time point. Controlling for confounders and interactions between variables, the model estimated that the odds ratio of using opioids after hospital discharge among those who received placebo was 5.67 times that of those who received FabAV. Patients who reported higher baseline pain, those with moderate as opposed to mild envenomation, and females were more likely to report opioid use at follow-up. Patients with ongoing limitations to functional status had an increased probability of opioid use, with a stronger association over time. Opioid use corresponded with the trial’s predefined criteria for full recovery, with only two patients reporting opioid use in the 24 hours prior to achieving full limb recovery and no patients in either group reporting opioid use after full limb recovery.

**Conclusion:**

In this study population, the proportion of patients using opioids for pain related to envenomation was smaller in the FabAV treatment group at all follow-up time points.

## INTRODUCTION

Between 5000 and 9000 persons in the United States (U.S.) seek treatment in an emergency department (ED) for snakebite each year.^1^ There were 2048 calls to U.S. poison control centers in 2016 from patients who experienced copperhead bites, of whom 1962 sought treatment in a healthcare facilty.^2^ There were close to another 2000 calls in which the type of snake was not identified.^2^ Severe manifestations of crotaline envenomation include coagulopathy, hypotension, shock and death; however, these are rare complications in copperhead envenomations.^3^,^4^,^5^

Essentially all copperhead envenomations cause local tissue injury, leading to inflammation, necrosis and endothelial damage.^6^,^7^,^8^ Edema and pain are the predominant symptoms that contribute to the morbidity of disease in the weeks following a copperhead envenomation.^6^,^7^,^8^ Expert consensus guidelines recommend avoiding aspirin and nonsteroidal anti-inflammatory drugs (NSAID) due to concern for bleeding, platelet dysfunction, and risk for prerenal toxicity.^9^,^10^ This leaves opioids as the current recommended means of pain control for patients suffering a copperhead envenomation.^9^,^10^

The current overuse and abuse of opioids has been declared an epidemic. The Centers for Disease Control and Prevention recently published guidelines to minimize the duration of opioid prescriptions.^11^ Many states have enacted legislation to limit prescriptions for opioid medications.^12^ Emerging data suggest that opioid prescriptions from the ED may contribute to long-term opioid use and potential abuse.^13^,^14^ Regardless of the cause for pain, increasing the duration of the initial opioid prescription decreased the likelihood that patients would discontinue opioids.^15^ Balancing the benefit of pain control and the risk of opioids, including potential for addiction, is clinically challenging.^16^

The primary objective of this secondary analysis was to describe post-discharge opioid use in subjects who suffered mild to moderate copperhead envenomation and were randomized to receive Crotalidae polyvalent immune Fab (ovine) antivenom (CroFab^®^, BTG International Inc., West Conshohocken, Pennsylvania) or placebo. A secondary objective was to explore the correlation between post-discharge opioid use and limb function recovery.

## METHODS

### Study Design

This study is a secondary analysis of a multicenter, randomized, double-blind, placebo-controlled trial of Fab antivenom (FabAV) vs placebo in patients with mild or moderate copperhead snake envenomation. The methods of this trial, including participant selection, randomization, treatment, and the full study protocol have been previously published.^17^ Use of opioid medications was defined as a secondary outcome measure during the design of the parent study. Patients aged 12 years or older with a mild or moderate copperhead envenomation to the distal arm or leg presenting within 24 hours of envenomation were randomized to receive FabAV or saline placebo. Mild bites were defined as swelling that crossed 0–1 major joints (wrist, elbow, ankle or knee) and moderate bites were defined as swelling that crossed two major joints. The randomization was stratified by severity (mild vs moderate), age (adult vs adolescent), and extremity affected (upper vs lower). Patients were identified and enrolled in the ED.

**Population Health Research Capsule**

What do we already know about this issue?

*Expert consensus guidelines call for avoidance of nonsteroidal anti-inflammatory drugs in patients suffering from copperhead envenomation. Opioids continue to be the recommended medication for their pain management*.

What was the research question?

Does treatment with Fab antivenom (FabAV) in patients who experience a copperhead envenomation affect opioid use?

What was the major finding of the study?

*The proportion of patients using opioids for copperhead envenomation pain was smaller in the FabAV group in comparison to placebo*.

How does this improve population health?

*The risk of iatrogenic addiction when prescribing opioids from the emergency department remains uncertain. This study suggests FabAV treatment decreases likelihood of opioid use after a copperhead envenomation*.

### Study Protocol

Patients randomized to FabAV received an initial dose of six vials in 250 milliliters (mL) of normal saline solution. A repeat dose of six vials of FabAV was administered to patients who failed to achieve initial control after the first dose. All patients were then administered two vials of FabAV at 6, 12, and 18 hours after initial control. Patients randomized to receive placebo received normal saline solution. Patients were treated by the emergency physician on duty upon presentation. Consult with poison control or a toxicologist was at the discretion of the treating physician. All other therapies administered to patients, including fluids, antiemetics, and pain medications, were at the discretion of the treating clinicians. Patients were admitted to the hospital or observed in the ED based on local hospital practice. Any medications prescribed at discharge were also decided upon by the treating clinician.

### Measures

The primary outcome measure for this secondary analysis was patient-reported, post-discharge opioid analgesic use during the 28 days following envenomation from a copperhead snakebite. We defined opioid analgesic use as an a priori secondary outcome of the initial study. Patients were evaluated on days 3, 7, 10, 14, 17, 21, 24, and 28 post-envenomation. At each post-envenomation time point, patients were asked to report all concomitant medication use. This included all analgesic medications used in the 24 hours prior to each assessment. They were asked specifically to report only on analgesic medications used to treat the pain associated with their snakebite. Analgesic use was classified as a dichotomous variable: “Opioid use” (including tramadol and combination products) and “No opioid use,” which included reported use of prescription analgesics (non-opioid), non-prescription analgesics, and no analgesics.

A secondary outcome for this study was to explore the relationship between the main trial’s primary measure of limb function recovery, the Patient-Specific Functional Scale (PSFS)^18^, and post-discharge opioid analgesic use. The PSFS was administered at all follow-up visits. This measure asked patients to report their ability to perform three self-identified important activities that they were unable to do or were having trouble doing as a result of their snakebite on a scale of 0 (“unable to perform activity”) to 10 (“able to perform activity at the same level as before injury or problem”). We calculated the mean score of all three activities, with larger values indicating more complete limb function recovery and a mean score of 10 indicating full recovery.

### Data Analysis

We conducted all statistical analyses on the modified intent-to-treat population. Two patients were excluded: one in the placebo group was lost to follow-up after discharge and did not have any post-discharge assessments completed; and a second in the treatment group who had falsified his snakebite was discontinued from the study by the treating investigator prior to unblinding of treatment arm. The patient with the falsified snakebite was included in the analyses for the parent paper, and was the only patient in the FabAV treatment group taking opioids at the 28-day follow-up. He was excluded from this analysis, as our primary goal was to determine whether FabAV for the treatment of copperhead snake envenomation affected use of opioids. The decision to exclude him from the secondary analysis was made prior to the design of the analysis plan.

We used summary statistics to describe characteristics of patients with any post-discharge, opioid analgesic use and those with no post-discharge opioid use. Additionally, the proportion of patients reporting opioid analgesic use was summarized for each follow-up time point, separated by treatment group. The differences between treatment groups are presented with exact 95% confidence intervals and p-values from a two-tailed Wald equivalence test.

To study the relationship between opioid use, functional status, and treatment with FabAV, we modeled the mean probability of opioid use across all visits using generalized estimating equations (GEE). A first-order autoregressive covariance structure was chosen to model the within-subject variance from visit to visit. The GEE model included effects for treatment group, visit number, PSFS score, envenomation severity (mild vs moderate), envenomation location (upper vs lower extremity), sex, age category (adolescent vs adult), time to treatment, and the interactions between treatment group and PSFS score, treatment group and visit number, and visit number and PSFS. We performed a backward stepwise regression and sequentially removed non-significant effects (defined as p>0.10 for interaction terms and p>0.05 for main effects). Once the model was finalized, each excluded term was added back separately to ensure that it did not drastically affect the results of the model, ensuring that there were no interactions unaccounted for in the final model. Results are presented as the mean probability of opioid use across all visits and the odds ratio of opioid use among patients treated with FabAV compared to those treated with placebo.

Missing values for opioid use and PSFS scores were imputed using the last observation carried forward method.

## RESULTS

### Demographics and clinical characteristics

We included 72 patients in this secondary analysis. Thirty-five (48.6%) patients reported use of an opioid at least once at a post-discharge, follow-up time point. A greater proportion of patients in the placebo group, those with moderate severity envenomation, females, and those patients with higher baseline pain scores reported post-discharge opioid analgesic use (Table 1). Rates of opioid use were similar between patients who suffered an upper extremity envenomation when compared to those who suffered a lower extremity envenomation.

### Primary outcome

**Figure 1:**
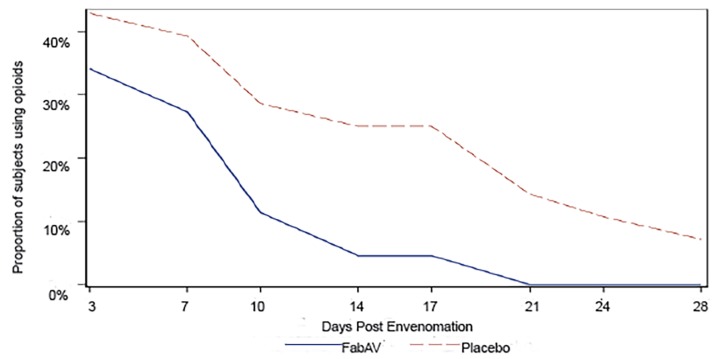
Proportion of subjects with mild or moderate copperhead envenomation who reported using opioid analgesics in the previous 24 hours. *FabAV*, Fab antivenom.

A greater proportion of patients treated with placebo reported opioid analgesic use at each follow-up time point compared to those treated with FabAV (Figure 1). All patients who experienced a copperhead snake envenomation and were treated with FabAV discontinued opioids by 21 days post-envenomation. There were patients within the placebo group who reported opioid use at each time point assessed (Table 2). Data from the main outcomes publication shows that there was opioid use in the FabAV group at day 28. In the main outcomes publication, Figure 1 does show that there was opioid use in the FabAV group at day 28.^17^ This represented one patient, who was one of the two patients excluded from this secondary analysis. This patient was found to have falsified a snakebite; thus, opioids taken would not have been used for treatment of pain associated with envenomation.

### Secondary outcomes

The final GEE model included the effects for treatment, visit number, PSFS score, the interaction between treatment and visit number, and the interaction between visit number and PSFS score. The model estimated mean probability of subjects using an opioid, after adjusting for time, PSFS, and the interaction between the two, was lower for the FabAV treatment group than the placebo group (Table 3). Overall, adjusting for other factors, patients in the placebo group were 5.67 times as likely to use an opioid post-discharge compared to patients in the FabAV group (p=0.008; Table 3).

In the GEE model, multiple variables were found to influence each other. The interaction between treatment and visit number was significant, indicating the effect of treatment on opioid use was dependent on time (p=0.028; Table 4). The estimated odds of opioid use was higher in the group treated with placebo at each time point, with increasing odds as time passed. At three days post-envenomation, the estimated odds of using opioids among the group treated with placebo was 1.38 times that of the FabAV group (95% CI 0.58, 3.27); by 14 days post-envenomation, the odds were 4.63 times for the placebo group in comparison to FabAV (95% CI 1.47, 14.62). The interaction between visit number and PSFS score was significant (p=0.042; Table 4). Patients with poor functional recovery, demonstrated by lower PSFS scores, were more likely to use opioids; this association also appeared stronger over time. At three days post-envenomation, PSFS score of one point lower was associated with a 1.08 times greater odds of opioid use (95% CI 0.97, 1.20). At 14 days post-envenomation, the odds were 1.29 times greater (95% CI 1.14, 1.47) and at 28 days post-envenomation the odds increased to 1.65 (95% CI 1.26, 2.17).

Finally, only two patients (both in the FabAV group) reported using opioids in the 24 hours prior to achieving full limb function recovery on the PSFS, and no subjects in either treatment group reported using opioids after reaching full recovery.

### Missing Values

Overall, only 3.5% of missing opioid use values were imputed. Within the placebo group, three patients had a total of eight imputed values; for the treatment group, six patients had a total of 12 imputed values.

## DISCUSSION

Although antivenom administration is the standard of care for rattlesnake envenomation, its use in copperhead envenomation has been controversial.^9^,^10^,^17^,^19^–^21^ This debate has been influenced by the low mortality associated with these bites, questionable effects on coagulation, the high cost of the drug, and the prior uncertainty about the efficacy for recovery from tissue injury.^20^, ^22^–^24^ There is also concern about allergic reaction to antivenom, although this is a rare occurrence since the ovine preparation used in FabAV was developed.^25^,^26^ A recent study did find that patients had earlier return of limb function following mild to moderate copperhead envenomation after treatment with FabAV.^17^ This same study found that subjects who were treated with FabAV also had lower pain scores and less opioid use than those subjects who were treated with placebo.^17^ The main argument against antivenom that now remains is one of cost vs benefit.^27^,^28^

It is often hard to predict how long patients will require pain control when they are seen at the time of initial injury. In general, patients with mild to moderate envenomation due to a copperhead bite have ongoing pain and swelling on average for about two weeks, although some have symptoms for prolonged periods of time.^6^,^7^,^17^,^29^ This secondary analysis found that less than half of all patients who suffered a copperhead envenomation required any use of opioids as an outpatient. Of note, those patients who were treated with FabAV had a decreased likelihood of opioid use and ceased use of opioids sooner than those subjects who received placebo.

There is no consensus in the literature about what initial duration or amount of opioid leads to addiction. In the opioid-naïve patient, it has been found that opioid prescriptions from the ED are associated with a lower risk of progression to long-term use than those prescribed in other settings.^30^ However, emerging data suggest that receipt of initial opioid prescriptions from the ED can contribute to long-term opioid abuse and addiction.^14^,^15^ There is a sharp increase in the probability of ongoing opioid use at one year when the initial prescription is for a duration of more than five days (hazard ratio for discontinued use one year after a 3–4 day supply 0.70; hazard ratio for discontinued use one year after a 5–7 day supply 0.48).^15^ This suggests that the risks of side effects from opioids and potential for long-term addiction should play a role in the decision-making process when considering treatment with antivenom following a copperhead envenomation. The risk and resultant cost of treatment for long-term addiction must also factor into any cost benefit analyses when discussing treatment with FabAV.

No current literature defines the number of patients who develop opioid use disorder after suffering a copperhead envenomation. Male gender and a younger age are both risk factors for the development of opioid use disorder.^32^ These same patient characteristics are associated with increased risk of unintentional snake envenomation.^33^,^34^ Although males are at higher risk for opioid use disorder, the rate at which females overdose on prescription opioids is much higher.^35^ In this study, we found that females were more likely to use opioids after a snake envenomation. While we do not know what the rate of opioid overdose might be in this specific patient population, any treatment that decreases the need for opioids, and therefore the risk for opioid dependence and addiction, should be considered.

There is another sharp increase in the probability of opioid use at one year when the initial prescription went beyond 30 days.^15^ Receiving a refill opioid prescription was also associated with an increased risk of ongoing opioid use at one year.^15^ In this secondary analysis, all copperhead snake envenomation subjects in the FabAV treatment arm had discontinued opioids by day 21, while 7% of the patients in the placebo group reported ongoing opioid use at 28 days post-envenomation. We did not follow patients beyond 28 days post-envenomation, but this raises concerns that the patients in the placebo group who continued to use opioids were at risk for development of opioid use disorder.

The parent study used the PSFS to report limb function disability following envenomation. This scale was initially designed to measure functional changes in patients with musculoskeletal disorders.^18^ It has since been validated in snakebite-envenomation patient populations.^7^,^29^,^31^ In this secondary analysis, we find that cessation of opioids correlates to an improvement in the PSFS. This further validates use of PSFS as a marker for patient recovery. It is especially concerning that the risk of opioid use with lack of improved PSFS scores, indicating ongoing disability, increased with time.

## LIMITATIONS

This study is a secondary analysis of data that were collected prospectively in a blinded fashion during the parent clinical trial. Use of opioid analgesics was defined as a secondary outcome measure a priori; this analysis was planned prior to any data review. However, the results should be treated as exploratory due to the design limitation. Future studies should focus on opioid use as a primary outcome and ultimately the impact of antivenom on opioid dependence consequent to snake envenomation. The latter will require large numbers and pragmatic designs.

These analyses relied on patient self-reported use of analgesics. We did not collect data on the doses that patients were taking or whether the medication was from an initial prescription or if they required a second prescription due to ongoing pain. Further study would be needed to determine whether non-opioid medications or non-pharmacologic means of pain treatment are as effective as opioids and whether they can be safely used to treat pain associated with copperhead envenomations.

The parent study did not limit use of concomitant treatments in the ED, such as pain medications, fluids or antiemetics. It is possible that use of these may influence patient experience of acute pain and need for ongoing treatment for pain after discharge. Additionally, opioid prescriptions were given at the discretion of the blinded treating physician and not determined by the study protocol. Simply receiving a prescription for opioids may influence whether patients take these medications.

This study only enrolled patients with a mild or moderate copperhead envenomation. As these envenomations tend to be less severe than those of other Crotalinae snakes, it is unclear whether opioid use would differ based on FabAV treatment for envenomations of other snake species. It is also unclear whether the more common systemic effects in other snake envenomations may affect the use and duration of opioid treatments. We were unable to perform subanalyses to determine whether the degree of envenomation (mild vs moderate) affected opioid use due to the low number of patients in the subgroups. This would be important to study if future studies enroll a larger number of patients.

## CONCLUSION

In a randomized, double-blind, placebo-controlled trial of Fab antivenom vs placebo, patients who received FabAV had a decreased likelihood of opioid use. Lower numbers on the Patient-Specific Functional Scale, indicating ongoing disability when compared to baseline, correlated with a greater probability of opioid use.

## Figures and Tables

**Table 1:** Demographic and clinical characteristics of subjects with mild or moderate copperhead envenomation, reported by use of post-discharge opioid analgesics.

	Any post-discharge opioid use N=35	No post-discharge opioid use N=37	Total N=72
Treatment, N (%)			
FabAV	18 (40.9%)	26 (59.1%)	44
Placebo	17 (60.7%)	11 (39.3%)	28
Age in years: mean (SD)	41.3 (15.95)	45.3 (19.28)	43.3 (17.73)
Age category, N (%)			
Adolescent	4 (50.0%)	4 (50.0%)	8
Adult	31 (48.4%)	33 (51.6%)	64
Baseline pain score*: mean (SD)	6.8 (2.57)	4.9 (2.83)	5.8 (2.85)
Envenomation location, N (%)			
Lower extremity	22 (48.9%)	23 (51.1%)	45
Upper extremity	13 (48.1%)	14 (51.9%)	27
Envenomation severity, N (%)			
Mild	29 (45.3%)	35 (54.7%)	64
Moderate	6 (75.0%)	2 (25.0%)	8
Gender, N (%)			
Female	19 (54.3%)	16 (45.7%)	35
Male	16 (43.2%)	21 (56.8%)	37
Hours to treatment mean (SD)	6.8 (5.06)	7.4 (5.53)	7.1 (5.28)

* Pain scores were evaluated using the 11-point numerical rating scale, with values ranging from 0 (no pain) -10 (worst pain ever).

*FabAV*, Fab antivenom; *SD*, standard deviation.

**Table 2:** Proportion of subjects with mild or moderate copperhead envenomation who reported using opioid analgesics in the previous 24 hours by time post-envenomation and treatment group.

Days from envenomation	Treatment	Opioid use (%)	Difference between treatment groups (95% CI)	P-value
3	Fab AV	34.1%	−8.8% (−31.8%, 14.3%)	0.456
	Placebo	42.9%		
7	Fab AV	27.3%	−12.0% (−34.4%, 10.4%)	.0293
	Placebo	39.3%		
10	Fab AV	11.4%	−17.2% (−36.4%, 2.0%)	0.079
	Placebo	28.6%		
14	Fab AV	4.5%	−20.5% (−37.6%, −3.3%)	0.020
	Placebo	25.0%		
17	Fab AV	4.5%	−20.5% (−37.6%, −3.3%)	0.020
	Placebo	25.0%		
21	Fab AV	0.0%	−14.3% (−27.2%, −1.3%)	0.031
	Placebo	14.3%		
24	Fab AV	0.0%	−10.7% (−22.2%, 0.7%)	0.067
	Placebo	10.7%		
28	Fab AV	0.0%	−7.1% (−16.7%, 2.4%)	0.067
	Placebo	7.1%		

*FabAV*, Fab antivenom; *CI*, confidence interval.

**Table 3:** Model estimated marginal mean probability of opioid analgesic use in subjects with mild or moderate copperhead envenomation.

Treatment group	Mean (standard error) probability of opioid use	Odds ratio	p-value
FabAV	0.036 (0.019)		0.008
Placebo	0.175 (0.047)	5.67 (1.57, 20.45)	

*FabAV*, Fab antivenom.

**Table 4:** Results of generalized estimating equations model of probability of opioid use in subjects with mild or moderate copperhead envenomation by fixed effect.

Effect	Chi-square	p-value
Treatment	1.83	0.177
Time (visit number)	0.14	0.707
PSFS score	1.77	0.183
Time *treatment interaction	4.84	0.028
Time *PSFS interaction	4.15	0.042

*PSFS*, Patient-specific functional scale.
